# Gene Therapy for Inherited Liver Disease: To Add or to Edit

**DOI:** 10.3390/ijms252312514

**Published:** 2024-11-21

**Authors:** Yue Chen, Niek P. van Til, Piter J. Bosma

**Affiliations:** 1 Amsterdam University Medical Center, Tytgat Institute for Liver and Intestinal Research, AG&M, University of Amsterdam, Meibergdreef 69-71, 1105 BK Amsterdam, The Netherlands; y.chen2@amsterdamumc.nl; 2 Amsterdam Leukodystrophy Center, Emma Children’s Hospital, Amsterdam University Medical Center, Amsterdam Neuroscience, Cellular & Molecular Mechanisms, Meibergdreef 9, 1105 AZ Amsterdam, The Netherlands; n.p.vantil@amsterdamumc.nl; 3 Department of Integrative Neurophysiology, Center for Neurogenomics and Cognitive Research, Vrije Universiteit Amsterdam, Amsterdam Neuroscience, De Boelelaan 1085, 1081 HV Amsterdam, The Netherlands

**Keywords:** inherited liver diseases, gene augmentation, genome editing

## Abstract

Patients suffering from an inherited severe liver disorder require lifelong treatment to prevent premature death. Until recently, the only curative treatment option was liver transplantation, which requires lifelong immune suppression. Now, liver-directed gene therapy, which is a much less invasive procedure, has become a market-approved treatment for hemophilia A and B. This may pave the way for it to become the treatment of choice for many other recessive inherited liver disorders with loss-of-function mutations. Inherited liver disease with toxic-gain-of-function or intrinsic hepatocyte damage may require alternative applications, such as integrating vectors or genome editing technologies, that can provide permanent or specific modification of the genome. We present an overview of currently available gene therapy strategies, i.e., gene supplementation, gene editing, and gene repair investigated in preclinical and clinical studies to treat inherited severe liver disorders. The advantages and limitations of these gene therapy applications are discussed in relation to the underlying disease mechanism.

## 1. Introduction

The liver, the largest solid organ in the human body, has a vital role in many metabolic, synthetic, and excretory processes—producing bile salts to enhance intestinal uptake of fat and fat-soluble vitamins, metabolizing and excreting toxic compounds via bile, producing blood components such as clotting factors [1] and albumin, and playing a role in cholesterol and triglyceride metabolism [2] and in regulating blood glucose levels [3]. As a consequence of these pivotal functions, if these genes are affected, this may not only influence liver function but can also inflict damage to other tissues such as blood, kidney, and brain. Although each of these hereditary disorders is rare, the total sum of patients suffering from an inherited liver disorder is considerable. For most inherited severe liver disorders, organ transplantation is still the only treatment option [4]. Although this highly invasive procedure can be curative, the need for life-long immune suppression to prevent organ rejection increases the risk of life-threatening infections, in addition to long-term adverse effects such as bone thinning, diabetes, diarrhea, high blood pressure, and high cholesterol [5]. In view of these complications, treatment of many inherited severe liver disorders is still an unmet medical need warranting the development of groundbreaking technologies, including gene therapy, that could potentially substitute liver transplantation in the future [6]. In this paper, we discuss gene therapy strategies and molecular advances to treat inherited liver disorders.

## 2. Adeno Associated Viral Vector Mediated Gene Augmentation In Vivo

Recessive inherited disorders, typically caused by loss-of-function mutations, can be treated by restoring protein function by expression of the encoding cDNA in the affected target tissue. Some typical applications of gene augmentation and gene editing are presented in Table 1. An advantageous property of targeting the liver and particular hepatocytes is that this can induce immune tolerance induction to the transgene product, making this an organ of choice for producing secreted proteins. In addition to blood components, such as clotting factors, the liver can produce and secrete enzymes to effectively correct lysosomal storage disease in several tissues. For these purposes, viral vectors are often used as the vehicles of choice because of their inherent properties to deliver genetic sequences efficiently. One commonly used viral vector system is based on adeno-associated virus (AAV), a replication-deficient nonpathogenic virus, which has become the method of choice for in vivo delivery of genes to the liver and other tissues. Besides the AAV serotype with liver and hepatocytes tropism, there are also liver-specific promoters, such as albumin (Alb), transthyretin (TTR), human alpha-antitrypsin (hAAT), that achieve high expression in hepatocytes.

The feasibility of this gene addition strategy has been established in numerous preclinical studies and has also been investigated in several clinical studies to treat inherited (liver) disorders (Table 2). The first successful correction using an AAV vector, delivered via the hepatic artery, was reported in 2006 [7]. In adult patients suffering from hemophilia B, a therapeutic level of factor IX expression was obtained for several weeks. A CD8+ T cell-mediated immune response towards the viral capsid caused rapid loss of transduced cells and correction, clearly demonstrating the need to overcome immunological hurdles for persistent therapeutic efficacy. Application of transient immune suppression, until the viral capsid has been degraded in hepatocytes, may have contributed to the long-term correction of factor IX deficiency in a subsequent trial [8]. Subsequently, proof of concept of AAV-mediated gene delivery for several inherited liver disorders has been established in pre-clinical models and small clinical trials (Table 2). The route to market approval of advanced therapeutic medicinal products (ATMPs) is expensive and generally takes longer than for conventional drugs. Market approval of AAV gene therapy has obtained for both hemophilia A (factor VIII deficiency) and hemophilia B (factor IX deficiency) in 2023 [9,10]. This demonstrates that gene therapy is fulfilling its long-term promise as a curative treatment option for genetic liver disorders.

However, demonstrating efficacy for these rare liver diseases is challenging due to the heterogeneity of symptoms, limited understanding of the natural disease course, and lack of predictive biomarkers. In view of safety, recombinant AAV (rAAV) is generally considered to have a low genotoxicity risk due to the lack of active integration into the host genome. A drawback related to this is the risk to lose episomal genomes and correction over time upon cell division, as reported in preclinical models [11,12]. This could compromise long-term term efficacy when applied in neonates and young children, particularly if there is chronic hepatocyte damage causing hepatocyte proliferation. For those diseases, strategies that support effective AAV re-administration may be beneficial to maintain sustained efficacy. Alternatively, integrating viral vectors, e.g., lentiviral vectors, or gene editing and gene repair technologies may be employed.

**Table 1:** Overview of liver gene therapy reported preclinical studies and clinical trials.

Gene Addition		
	Preclinical Studies	Clinical Trials
Hemophilia A *	AAV2-cFVIII, AAV6-cFVIII and AAV8-cFVIII, mice and dogs [13]; BMN 270 (AAV5-hFVIII-SQ, valoctocogene roxaparvovec), mice and nonhuman primates (NHPs) [14].	NCT02576795 funded by BioMarin Pharmaceutical (AAV5-hFVIII-SQ) [15]; NCT03003533 funded by Spark Therapeutics (novel AAV) [16]; Valoctocogene Roxaparvovec, phase 3 in 134 male patients [17].
Hemophilia B **	Several pre-clinical studies were performed. In mice, e.g., [18]; in dogs, e.g., [19]; and in nonhuman primates, e.g., [20].	Several studies in small groups of adult patients were performed. The first trial (NCT00076557) resulted in a transient correction [7]. Upon showing efficacy of transient immune suppression in a second trial (NCT03369444), long-term correction was obtained, Nathwani et al., 2011 [8]. Additional studies have further optimized the effect, e.g., by using the factor IX Padua mutant with higher activity. For a complete overview, see Muczynski et al., 2024 [21].
Crigler–Najjar Syndrome	Several preclinical studies were performed in rats, e.g., [22], and in mice, e.g., [23].	The first trial (NCT03223194) only included a single patient and resulted in a transient correction. The second trial NCT03466463 treating 5 adult female patients did show sustained correction when using the higher dose, D’Antiga et al., 2023 [24].
OTCD	Several AAV serotypes (2, 7, 8 and 9 resp.) expressing OTC were studied in mice, showing prolonged correction in adult animal, [25], but transient correction in neonatal mice [26,27]. In primates, an AAV8 (DTX301) vector was tested in macaques [28], and an AAV vector with a modified capsid, AAVLKO3.hOTC, was tested in juvenile cynomolgus monkeys [29]. AAV2/8-hOTC-CO was tested in mice [30].	scAAV8OTC, phase 1–2, ongoing [31] Atrial using an Ad5vector NCT00004386 was unsuccessful and was stopped after the death of a patient. A phase 1–2 trial (NCT02991144) using scAAV8-OTC in adults reported correction [32]. A phase 3 trial (NCT05345171) included patients > 12 y. A trial in babies less than 9 months old is ongoing (NCT06255782).
GSD-Ia	The liver is used to produce G6PC to overcome enzyme replacement therapy. Firstly in mice [33], then secondly, a similar approach in mice and dogs [34].	A phase 1–2 trial using AAV8-G6PC was completed (NCT03517085) and, based on results, a phase III trial including patients > 8 y is now ongoing (NCT05139316).
Mucopolysaccharidosis, different diseases	In mice, e.g., Watson et al., 1998 [35], reporting correction and Cardone et al., 2006 [36]; in cats, e.g., Cotugno et al., 2011 [37]; in NHPs, e.g., Hordeaux et al., 2019 [38].	Many in vivo strategies using AAV6, AAV8, AAV9, and AAVrh10 vectors [39].
AIP	Mouse model for porphobilinogen deaminase (PBGD)-deficiency rAAV2/5- hPBGD, mice [40]; rAAV5-cohPBGD, safety and efficacy in macaques [41].	rAAV2/5- PBGD, phase 1 in 8 patients (NCT02082860) [42].
AATD	To correct liver deficiency and the tox of the misfolded enzyme, a combination was used: knock-down misfolded form and expression of the correct form in mice, Li et al. [43]; miRNA and gene addition, Mueller et al. [44]; Most clinical trials targeted muscle, because the mutated allele causes liver damage, efficacy of gene editing to disrupt this allele is investigated [45,46].	rAAV2-CB-hAAT, phase 1, (NCT01054339) [47] muscle targeted; rAAV2-AAT, phase 1 in 12 patients, (NCT00377416) [48] muscle targeted, safe but not effective; rAAV1-CB-hAAT, phase 2 in 9 patients, (NCT00430768) [49] muscle targeted; recent trial targeting liver (NCT02168686) (AAVrh10), no data reported; good candidate for gene editing. Maybe combined with gene addition. Fazirsiran to reduce misfolded enzyme [50].
Wilson disease	AAV8-TTR-hATP7Bco, mice [51].	AAVx-ATP7B, phase 1, ongoing (NCT04884815).
HoFH	AAV2-TBG-hLDLR, AAV2/7-TBG-hLDLR, AAV2/8-TBG-hLDLR, mice [52]; AAV8.TBG.mLDLR, AAV8.TBG.hLDLR, mice [53]; AAV8.IVS2.hLDLR011-T, mice [54]; AAV8.TBG.hLDLR, macaques [55].	AAV8-hLDLR, phase 1–2 in 9 patients (NCT02651675). Results not reported but discontinued. Another phase 1 trial (NCT06125847) is ongoing.
PFIC3	AAV8-hABCB4, mice [56]; AAV-MDR3-Aco, mice [57]; AAV8-MDR3, mice [58].	N.A.

* Hemophilia A: Roctavian^®^, the AAV5 SQ product was approved by EMA [59] in 2022 and approved by FDA in 2023 [60]. ** Hemophilia B: Hemgenix^®^, AAV5, factor IX-Padua was approved by EMA in 2023 [61] and approved by FDA in 2022 [62]. OTCD, ornithine transcarbamylase deficiency; GSD-Ia, glycogen storage disease type Ia; AIP, acute intermittent porphyria; AATD, alpha 1-antitrypsin deficiency; HoFH, homozygous familial hypercholesterolemia; PFIC3, progressive familial intrahepatic cholestasis type 3.

**Table 2:** An overview of typical applications of gene augmentation and genome editing technologies.

Application of Gene Augmentation	Application of Genome Editing
To restore functional protein expression for loss-of-function mutations	To correct loss-of-function and knock-out toxic-gain-of-function mutations
To produce enzymes in the liver that upon secretion are delivered to other tissues, i.e., lysosomal enzymes	To correct mutations in genes that need tight control by their endogenous promoter
	To modify a metabolic process, i.e., knocking out PCSK9 expression to lower serum cholesterol

### AAV Vector Limitations

Achieving lifelong therapeutic efficacy is essential for patients suffering from inherited disorders, for which effective initial rAAV hepatocyte transduction is paramount. Long-term AAV vector expression is feasible, as has been demonstrated by stable plasma factor IX levels in adult hemophilia B patients treated > 13 years earlier. Although data on durability of FVIII expression in patients suffering from hemophilia A are still being collected, these do indicate long-term correction is less predictable. While modeling of activity data over time does indicate factor IX expression will be at a therapeutic level after 25 years after AAV gene therapy in 80% of all patients, factor VIII activity seems less stable, and many patients may need to start administering this clotting factor after AAV gene therapy before this time [63].

Despite these successes, there are some obstacles to effectively treating these patients. First, prior natural AAV infection, which occurs in the majority of the human population, results in neutralizing antibodies. For instance, the existence of this immunity towards AAV made the use of rAAV-8 in ~1/3 of adult Crigler–Najjar syndrome patients ineligible [64]. Depending on AAV serotype and country, neutralizing antibodies are found in 60–90% of all adults [65]. A second obstacle are immune responses developed against the rAAV capsid after dosing. A third problem that may occur is loss of lifelong therapeutic correction due to hepatotoxicity or infections that accelerate hepatocyte proliferation. This will lead to the loss of episomal AAV vector genomes, thereby compromising long-term efficacy. Even under normal physiological conditions, the low basal level of hepatocyte proliferation could result in a gradual loss of rAAV copies per cell over time [66]. Furthermore, in children, and even more so in neonates, liver growth provides a higher basal level of hepatocyte proliferation that may compromise lifelong rAAV efficacy. Several preclinical studies in neonatal liver disease models have demonstrated loss of efficacy during aging. For instance, Cunningham et al. found AAV2/8-mediated correction of ornithine transcarbamylase deficiency in adult spfash mice is stable for life, but in neonatal spfash mice, the correction declined rapidly in 4 weeks [26]. In two animal models for Crigler–Najjar, this reduction of correction due to loss of AAV genomes has also been shown [12,67].

Consequently, developing effective methods for rAAV vector re-administration to ensure lifelong efficacy is paramount. This is particularly complicated by strong immune responses primed by prior rAAV administration. The presence of these neutralizing antibodies (NAbs) against AAV can bind the rAAV and abrogate hepatocyte transduction. Different strategies have been pursued to prevent the production of these NAbs mounted by the initial administration. Preclinical studies recently showed that immune suppression during exposure of the vector capsid to B cells prevented high titer-neutralizing antibodies [68]. In the natural rat model for Crigler–Najjar, however, immune suppression did prevent NAbs after the first injection, but a high titer was induced upon re-treatment, suggesting the NAbs response could only be prevented in a naïve immune system not previously exposed to rAAV [12]. Since AAV is endogenous in humans, infection will occur in most patients, resulting in a gradual increase in the prevalence of pre-existing immunity towards AAV with age [65,69]; this may complicate re-administration as well. Therefore, techniques to remove NAbs after they have occurred may also be needed. A promising strategy is proteolytic degradation of IgGs for effective re-administration [70]. Nevertheless, removal of IgGs, combined with immune suppressive treatments, may render patients more susceptible to recurrent infections. In that respect, specific removal of anti-AAV IgGs would be a safer option [71]. Although these strategies still await testing in clinical trials, the nonclinical progress made indicates that lasting therapeutic efficacy of AAV-mediated gene addition may be possible in the future.

Depending on the specific genetic defect that causes the inherited liver disease, different levels of hepatocyte transduction may be required. If a small percentage of normal activity provides therapeutic efficacy, low hepatocyte transduction percentages (<10%) can be sufficient [7,72]. For therapeutic correction, currently used doses (range 0.5–2 × 10^13^ vg/kg) using AAV serotypes 5 or 8 are sufficient to stop or reduce factor IX administrations to stop bleeding episodes in patients suffering from hemophilia B or to stop daily phototherapy in patients suffering from Crigler–Najjar syndrome. However, for correction of inherited liver disease that requires higher transduction efficiencies (10–100%), 10–100-fold higher rAAV vector doses are needed. Administration of these high rAAV doses can cause acute liver toxicity and liver failure, as observed in patients with neurological or muscle disorders [73]. This is a limitation of currently available rAAV vectors, which may leave liver diseases like progressive familial intrahepatic cholestasis (PFIC) currently out of reach for potential treatment because it requires correction of the majority of hepatocytes (>90%). In another mouse model for *Abcb4* deficiency, a model for PFIC3, a 100-fold higher AAV8 vector dose (5 × 10^13^ vg/kg) was needed for therapeutic correction compared to the 5 × 10^11^ vg/kg to correct *Ugt1a1* deficiency. Although this applied dose of AAV8-ABCB4 did result in prolonged correction in treated *Abcb4^−/−^* mice, the expression of human ABCB4 expression was not established in all hepatocytes. The ongoing loss of *Abcb4^−/−^*-deficient hepatocytes caused continuous compensatory proliferation of hepatocytes, including that of transduced hepatocytes, resulting in a gradual loss of rAAV vector genomes over time, from approximately 50% at week 10 to 30% at week 26 after treatment, and consequently loss of correction [56]. PFIC3 patients display even more severe liver pathology than the *Abcb4^−/−^* mouse model, prompting more damage to nontransduced hepatocytes, a higher proliferation rate, and risk of a more rapid loss of efficacy, as demonstrated in an *Abcb4^−/−^* mouse with a more severe phenotype [57].

AAV capsid engineering may further improve hepatocyte transduction efficiency at a lower dose and reduce the uptake by other liver cells, such as macrophages [74]. Additionally, removal of CpG motives in the vector genome, which are recognized by Toll-like Receptor 9 (TLR9), may also reduce innate immune responses. The relevance of genome modifications to lower innate immunity has been demonstrated, for instance, in its effect on the neuronal structure in mice [75]. Whether these types of abovementioned modifications, as well as optimization of rAAV manufacturing to prevent empty capsids, will increase therapeutic efficacy in the liver is still under debate [76,77].

Finally, a limitation of AAV packaging capacity is the maximum 4.7 Kb of DNA. For genes having a larger coding region, such as numerous liver-specific transporters, efficient packaging will be compromised, resulting in low titers. Although dual AAV vectors have been developed to overcome the relatively small packaging capacity, their efficiency is reduced significantly [78].

## 3. Lentiviral Vectors for Gene Augmentation In Vivo

For inherited diseases such as PFIC1, PFIC2, PFIC3, and Wilson’s disease, the coding regions of the *ATP8B1*, *ABCB11*, *ABCB4*, and *ATP7B* sequences exceed the AAV vector capacity. Viral vectors that have a larger packaging capacity, such as third-generation self-inactivating lentiviral vectors (~8 Kb), may be more suitable [79,80]. Another advantage would be their active integration into the host genome, which will provide persistent and stable correction, which is not lost upon hepatocyte proliferation. This could maintain corrected hepatocytes during liver growth, as has been demonstrated by providing stable FIX expression using low-dose UCOE-FIX vectors delivered in prenatal mice [81]. In addition, it could also promote gradual hepatocyte repopulation of the liver if the corrected hepatocytes have a selective growth advantage in liver disease with endogenous hepatocyte damage, such as in hereditary tyrosinemia type 1 (HT1). In two HT1 pigs treated with fumarylacetoacetate hydrolase (FAH) lentiviral vectors in vivo, extensive liver repopulation of FAH-positive hepatocytes had occurred, totaling 69% and 78% of the total number of cells in the liver, respectively, at 225 days post-treatment [82].

However, the low hepatocyte transduction efficiency of lentiviral vectors upon systemic administration in vivo has delayed its use for clinical application of liver-directed gene therapy. Vector improvements, such as incorporation of a microRNA (miR) target sequence against miR-142, restricting the expression of the therapeutic protein to hepatocytes and preventing expression in antigen-presenting cells, resulted in stable long-term Factor IX (FIX) plasma levels in dogs [83]. Nonetheless, the FIX plasma levels were low, <2% of normal activity compared to the 5–10% upon a moderate AAV-2 dose (4 × 10^11^) administration in dogs [84]. Additional vector modifications, especially preventing phagocytic uptake by including the ‘do-not-eat-me’ protein CD47 in viral particles, robustly improved the hepatocyte transduction efficiency 10-fold in mice and achieved a 3-fold higher stable FIX plasma levels in nonhuman primates [85,86]. These modifications, together with the enlarged packaging capacity and stable integration into the host genome, may enable the exploitation of lentiviral vectors to treat liver diseases that cause liver damage, such as PFIC1, PFIC2, and PFIC3. In a previous study in *Abcb4^−/−^* mice, we showed that healthy hepatocytes expressing endogenous *Abcb4* protein resulted in efficient repopulation of the liver [87], but because lentiviral particles are enveloped, membrane lipid compositions may affect titers significantly [88]. In this particular case, vector design may be further adjusted to prevent expression of the therapeutic protein during lentiviral manufacturing. In vivo lentiviral vector gene therapy may therefore be more suitable for clinical application for diseases that need a low percentage of hepatocyte correction, such as hemophilia. A disadvantage of integrating vectors, such as lentiviral vectors, is that there is a risk of genotoxicity, although this is expected to be low; hepatocytes, most of the time, are quiescent and nondividing, and as such generally the likelihood of genotoxicity is less [89]. Nonintegrating lentiviral vectors were also explored [90] and showed to induce immune tolerance and sustained FIX expression, but AAV vectors may then be preferred to obtain improved efficacy.

Ex vivo gene therapy was also explored in preclinical models for liver disease. This approach could also reduce potential off-target transduction and associated side effects, because hepatocytes could be directly genetically modified. Ex vivo lentiviral vector gene therapy was successfully shown to stably transduce hematopoietic stem cells (HSC) in clinical trials to correct metabolic diseases [91,92]. In contrast to autologous HSC, which can be retrieved through apheresis from mobilized blood relatively easily, hepatocyte retrieval and transplantation is a complex and invasive procedure. Furthermore, the grafting efficiency of corrected hepatocytes in the liver is low. This explains why ex vivo gene therapy has only been effective in animal models with liver diseases causing severe hepatocyte damage [87,93], but this approach is less likely to be successful for treating genetic liver diseases. An overview of the different properties of AAV vectors and lentiviral vectors is shown in Table 3.

## 4. Gene Editing Technologies

In contrast to loss-of-function mutations, in which gene addition can restore deficiencies, pathologic dominant gain-of-function mutations cannot be treated effectively using gene supplementation. These dominant disorders will require direct genomic correction to be effective. Furthermore, genome editing also induces permanent genomic corrections, which is beneficial for genetic liver diseases with intrinsic hepatocyte damage.

Various genome editing technologies were developed in the last three decades. One of the first programmable nucleases are the zinc finger nucleases (ZFN), developed in 1996 by combining a DNA-binding zinc finger domain with the nuclease domain of the restriction enzyme FokI [94]. Subsequently, other options were developed, like meganucleases in 2003, based on homing endonucleases found in phages, bacteria, and various eukaryotes [95]. In 2010, transcription activator-like effector nucleases (TALENs) were developed [96]. TALENS consist of the DNA-binding domain of a plant pathogen Xanthomonas sp. and Fok1 nuclease domain. Although all three allow targeting to specific DNA sequences, their construction is complicated and time consuming. The invention of clustered regularly interspaced short palindromic repeats (CRISPR)/Cas9 made targeted genome cutting much easier [97,98]. This flexible system is based on a bacterial adaptive immune defense consisting of a surveillance complex guided by two RNA molecules [99,100]. A trans-activating crRNA (tracrRNA), base pairs with the repeat sequence in the CRISPR-RNAs (crRNAs), forming a dual RNA hybrid structure guiding Cas9 to cleave any DNA containing a complementary 20-nucleotide (nt) target sequence and adjacent protospacer adjacent motif (PAM), a short sequence conserved for each Cas enzyme. The finding that the crRNA and tracrRNA could be combined into a single guide RNA (sgRNA) simplified this mechanism and resulted in a highly flexible gene modification tool [97]. By modifying the guide RNA, the Cas9 nuclease can be targeted to a specific sequence. The target sequence is recognized by the sgRNA, which can easily be modified and targeted to most genomic sequences. To trigger a double-strand break (DSB), the presence of the PAM sequence is essential. These PAM sites are conserved for each Cas enzyme, i.e., NGG for spyCas-9 and NNGRRT for SauCas9, which limits the flexibility to which genomic sequences can be targeted. The location of the double spread break is related to the PAM, for instance, three bases upstream of the PAM in the case of Cas9 [101]. More genome engineering enzymes of the Cas protein family have been discovered, such as Cas9, Cas12, Cascade, and Cas13 orthologs, broadening genome accessibility; because each enzyme has its own unique characteristics, this resulted in a flexible toolbox to target any genomic DNA sequence [102] (Table 4).

### 4.1. Genome Editing to Eliminate Endogenous Expression

The double-strand breaks induced by these targeted nucleases result in the activation of DNA repair mechanisms. In somatic cells, like hepatocytes, double-strand breaks are mostly repaired by nonhomologous end joining (NHEJ), an error-prone mechanism. The NHEJ directly joins both ends flanking the double-strand DNA break, often resulting in nucleotide insertion or deletion (indels), causing frame-shift mutations, introducing premature stop-codons leading to nonsense-mediated RNA-decay eliminating gene expression. For some diseases, gene knock-out could be an effective therapeutic strategy that is now explored in clinical trials—for instance, knocking out the binding sites for the lymphoma-related factor (LRF) repressor in the γ-globin promoter to reactivate the expression of fetal hemoglobin using zinc finger nucleases (NCT03432364) or CRISPR (NCT03655678) to treat β-thalassemia [112], or cancer immunotherapy to eliminate endogenous expression of selected genes in T cells [113]. This approach may also be an option to remove pathological nucleotide repeats in, for instance, Huntington’s disease [114]. Recently, CRISPR/Cas9 mRNA with a gRNA delivered using lipid nanoparticles was used to reduce kallikrein B1 expression in the liver, resulting in effective treatment of hereditary Angioedema [115].

The downsides of generating DSB by nucleases are potential genotoxic effects, such as large deletions and chromosomal translocations [116]. Using nickases, which are Cas nucleases in which one of the nuclease domains has been inactivated, generates single DNA strand breaks. Especially employing double or paired nickase approaches, efficient gene inactivation can be established with a lower genotoxic risk [117].

### 4.2. Gene Correction Through Homology-Directed Repair

In contrast to NHEJ, homology-directed repair (HDR) is a precise repair mechanism that uses DNA stretches with sequences homologous to the targeted region to correct DNA damage. This mechanism is highly active during the meiosis, while in all other situations it is less active than NHEJ. To use this approach effectively, DNA templates encoding the correct sequence overlapping the genomic mutation flanked by two homologous arms are needed [118]. The length of the repair template, having two homologous arms of around 900 bp, was around 2 Kb. Since DNA templates longer than 200 bp do require a viral vector, such as AAV, for efficient in vivo delivery, this approach faces the immunologic hurdles discussed previously. In pre-clinical models, this strategy has been applied successfully. It resulted in efficient correction, especially in case there was a survival benefit of the corrected hepatocytes, stimulating a gradual repopulation of the liver by the corrected cells [46,119]. In this mouse model, the misfolded alpha1-antitrypsin induced endoplasmic reticulum stress, resulting in hepatocyte damage. Repair of the missense mutation restored protein folding, reduced ER stress, and improved viability of corrected hepatocytes compared to noncorrected cells.

Although the efficiency of HDR in adult liver is low, <0.5% of the hepatocytes targeted insertion of a reporter gene, whereas in neonatal liver, targeted insertion was obtained in 5 to 10% of the hepatocytes [120]. Although effective delivery of a repair template does require a viral vector, with all the challenges mentioned earlier, the possibility to insert a gene in the genome in a safe region is a major advantage. Furthermore, since correction of a small percentage of hepatocytes provides therapeutic efficacy for several inherited liver diseases, this strategy seems a feasible option. Selecting the appropriate genomic region to insert an expression cassette or exploit endogenous expression is important. A successful approach is targeted insertion at the end of the albumin open reading frame. The albumin gene is highly expressed in the liver, and this resulted in effective secretion of the therapeutic proteins in pre-clinical models for hemophilia A and B and lysosomal storage diseases like Fabry and Gaucher [121,122]. For diseases requiring intracellular expression, this approach has been tested successfully, resulting in partial correction in a mice model for Crigler–Najjar syndrome [123]. Zinc finger nucleases have been used to target therapeutic genes in the albumin locus in patients suffering from mucopolysaccharidosis 1/II or hemophilia B. This complicated strategy required administration of three AAV vectors simultaneously, one containing the donor template and the other two each encoding a zinc finger, and did not result in detectable therapeutic efficacy [124]. Improved and timely delivery of all components and enhancing HDR activity may be needed to restore expression in a sufficient number of hepatocytes to obtain therapeutic efficacy in adults [125]. Another problem observed in preclinical trials is potential off-target genome editing. The specificity of HDR-mediated correction by nickases instead of nucleases, such as a double-nickase strategy, reduced off-target editing significantly. This almost completely prevented indels and increased the precise editing frequencies to ~90% in vitro in cultured cells [126]. Although this approach is promising and appears to be a feasible option to treat inherited liver disease, it does not exclude introducing aberrations, including large deletions, at the targeted site. A major complication of HDR is the efficient delivery of a DNA repair template, currently only feasible using a viral vector like AAV, with the immunological challenges mentioned earlier.

In conclusion, HDR approaches seem a feasible treatment option for recessively inherited liver diseases for which a relatively low restoration of function already leads to sufficient therapeutic benefit.

### 4.3. Base Editing to Restore Gene Function

**Figure 1:**
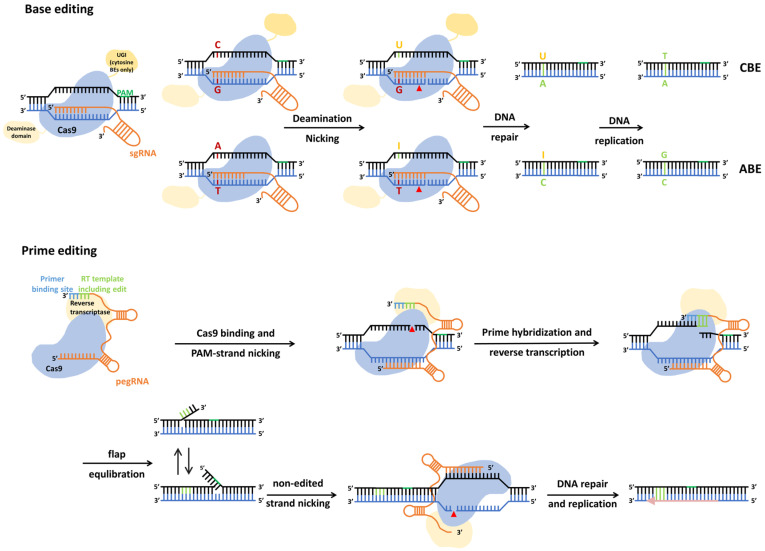
Base editing and prime editing systems.

The flexibility of genome editing tools has been used to target DNA-modifying enzymes to specific genomic regions. For instance, a dead Cas9 protein with mutated nuclease domains fused to a DNA methylase, has been applied to introduce targeted DNA methylation to repress gene expression [127]. This approach has also been adapted to generate base editors (BE) for direct editing of pathologic point mutations in the genome in vivo (Figure 1).

Base editing: A gRNA targets the Cas9 nickase fused to cytosine or adenosine deaminase to the missense mutation. The cytosine deaminase activity converts a cytosine into a uracil; the adenosine deaminase converts an adenosine into an inosine. Correction by the DNA miss-match repair system results in the editing of the missense mutation.

Prime editing: pegRNA targets Cas9 nickase fused to reverse transcriptase to the mutated sequence and provides the sequence for the reverse transcriptase to generate the repair on site. The edited 3′flap is copied to the nonedited strand.

For both methods, an additional single guide RNA (sgRNA) to introduce a nick to the nonedited strand will enhance editing efficiency.

CBE, cytosine base editing; ABE, adenine base editors; UGI, uracil DNA glycosylase inhibitor; RT, reverse transcriptase; PBS, primer binding site; pegRNA, prime editing guide RNA; sgRNA, single guide RNA; PAM, protospacer-adjacent motif. Adapted from Matsoukas 2020 et al., Rattananon et al., and Liu 2022 et al. [128,129,130].

BEs consist of a nCas9 nickase fused to a cytidine deaminase, converting cytidine into uracil base pairing as a thymidine, or an adenosine deaminase, converting an adenosine into an inosine base pairing as a guanosine. By targeting both strands, these enzymes can convert a C to T, G to A, A to G, or T to C. Fusion of these deaminase to nCas9, with the missense mutation (H840A), results in a flexible enzyme that can mutate these bases in the genome [131,132]. As discussed, replacing nuclease with a nickase significantly reduces the risk of genotoxicity caused by double-strand DNA breaks. Many pathogenic mutations are caused by a single nucleotide change, and in recessive inherited diseases, correction of one allele will prevent disease. A limitation is the inability to change each nucleotide into one of the other three options; changes that are not possible are C to A, C to G, G to C, G to T, A to C, A to T, T to A, and T to G. The specificity of BEs also needs to be improved because bystander edits are known to occur due to the activity of deaminase in the vicinity of the target sequence. This could nullify mutating the specific nucleotide or potentially create even more severe pathogenic variants due to newly acquired missense mutations [133,134].

A recent pre-clinical study showed the feasibility of the effective lipid nanoparticle delivery of a base editor in nonhuman primate (NHP), knocking-out the expression of the proprotein convertase subtilisin/kexin type 9 (PCSK-9). This negative regulator of the low-density lipoprotein (LDL) receptor is primarily expressed in the liver, and upon knocking out, serum cholesterol levels were reduced due to increased LDL-r expression [135,136]. In contrast to strategies aimed at correcting a single nucleotide missense mutation, bystander edits should not be a major concern for this knock-out strategy. The promising data in the NHP resulted in starting of a phase 1 trial in patients suffering from high cholesterol due to heterozygous familial hypercholesterolemia (NCT05398029). This strategy seems to be a feasible and safe approach for liver disorders in which abolishing gene expression would provide a treatment [137].

These complexes can be delivered by viral vectors but also by mRNA-mediated delivery, which excludes the unwanted insertion of BE sequences in the host genome that can result in prolonged BE expression and cause potential genome toxicity. Furthermore, mRNA complexes do not need to enter the nucleus, so nonviral lipid nanoparticles (LNPs) are considered an appropriate vehicle for efficient delivery to the liver. However, the design of LNPs and controlling biodistribution to prevent undesired off-target effects in other tissues remain a challenge for this promising technology [138,139]. This approach may allow repeated dosing to reach therapeutic efficacy.

### 4.4. Prime Editing to Restore Gene Function

Another more recently developed tool are prime editors (PEs). In contrast to HDR, PEs do not rely on generating a DSB, thus having a reduced risk for chromosomal translocations, and do not require an exogenous donor DNA repair template. PEs are not restricted to specific nucleotide conversions like base editors and can correct all missense mutations. Their flexibility may further be increased by using different Cas proteins to overcome the limitation imposed by the requirement of a specific PAM-sequence near the target sequence [140]. PEs consist of Cas9 nickases fused to an engineered reverse transcriptase (RT) to generate the repair template on site. This allows the donor-free precise DNA editing of virtually all missense mutations, as well as frameshift mutations, by inserting or deleting one-to-several nucleotides. The PE guide RNA (pegRNA) not only specifies the target site but also encodes the DNA repair template to correct the pathogenic mutation (Figure 1). The nCas9 of PEs generates a single-strand nick, resulting in a 3′-single-stranded DNA (ssDNA) strand. This hybridizes to the primer binding site of the pegRNA functioning as a start for the RT to copy the correct sequence encoded by the pegRNA into a DNA repair template on site. This results in two redundant ssDNA flaps: a 5′flap, containing the unedited sequence, and a 3′flap, containing the edited sequence. The inherent susceptibility of 5′flaps to excision by endogenous structure-specific endonucleases leads to hybridization of the edited 3′flap. Upon ligation, the resulting hetero duplex is recognized by the DNA mismatch repair (MMR) processes. To increase the incorporation of the desired modification, a guide RNA targeting the unedited strand is added. The nick in the unedited strand, generated by the Cas nickase, increases the selection of the edited strand as a template by the MMR, resulting in the desired editing [141,142].

Prime editing has been tested in several cell types and preclinical animal models such as the PiZ transgenic mouse model of alpha1-antitrypsin deficiency (AATD). In this mouse, a G-to-A inactivating mutation is present in the serine protease inhibitor peptidase inhibitor family member 1 (SERPINA1) [130]. Using hydrodynamic tail vein injection delivery of plasmids encoding PE, 6.7% editing was obtained for the optimized construct. This percentage of correction would be sufficient for diseases for which <10% of normal activity can be therapeutic. With delivery using the clinically applicable dual AAV vector delivery method needed to contain the 6.3 Kb long PE coding region, less than 1% correction was obtained. Optimization of PE delivery methods therefore seem needed to ensure effective gene repair in patients. Although PE applications have not yet entered clinical trials, studies in preclinical animal models show proof of concept and further analysis will generate more insight into the risks of off-target effects [143,144,145]. In view of their limited efficacy, this form of gene repair seems best suitable for liver diseases causing continuous hepatocyte damage. Repair of the pathogenic mutation in a small percentage will result in gradual repopulation of the liver by the corrected hepatocytes. Compared to base-editing, current PEs are less efficient. Novel PEs and pegRNAs with improved efficiency have been generated, and insertion of longer DNA templates has also been demonstrated, rendering this flexible strategy a promising method to treat inherited liver disorders [146]. Future nonclinical and clinical studies should generate more insight into the efficacy and off-target editing and will set the foundations to improve PEs for future applications.

### 4.5. Limitations of Genome Editing In Vivo

Although founder effects are present, for most inherited disorders, the pathogenic mutations are very heterogeneous. For instance, for Crigler–Najjar syndrome type I, the most severe form, 61 different mutations are listed in the Human Gene Mutation Database (https://www.hgmd.cf.ac.uk, accessed on 5 November 2024). Targeting a base editor or a prime editor requires a mutation-specific guide RNA for each pathogenic mutation, which may only be present in very few patients. Development of such a ‘tailor-made’ therapy will therefore be expensive and therefore will only be applicable to founder mutations present in a larger number of patients. Current research will increase the knowledge for pegRNA efficacy and their on-and off-target effects. This will result in improved algorithms for guide design. Prediction tools that are utilized to assess, for instance, pegRNA structure and function, which may incorporate artificial intelligence, may aid in future design to target each specific mutation. Regulatory agencies will need to adapt genome editing regulations to expedite use of these technologies in the clinic.

Gene augmentation may therefore remain the preferred strategy for diseases such as hemophilia A and B. In view of showing efficacy in small numbers of patients and with respect to cost effectiveness, gene addition currently seems a more realistic strategy to provide a cure for recessive inherited liver disorders. The insertion of therapeutic genes in a safe locus could become the optimal strategy. This integrating approach will also be applicable to diseases causing continuous liver damage and in neonatal patients when hepatocytes are proliferating. A major advantage of this strategy is that for each recessive inherited liver disorder, a single therapeutic construct can provide correction in all patients irrespective of their specific mutation [147].

In contrast, for disorders with a dominant inheritance pattern, gene deletion or repair may be the preferred option. Furthermore, in toxic gain-of-function mutations that cause hepatocyte damage, the survival benefit of the corrected hepatocytes will result in gradual repopulation of the liver. In view of the current low efficiency of these approaches, repopulation seems to be needed for therapeutic efficacy.

## 5. Conclusions

The market registration of AAV-mediated gene therapy for hemophilia A and B demonstrates the feasibility of gene supplementation for treatment of inherited liver disorders that require a relatively low hepatocyte transduction efficiency (<10%) to be curative. If higher correction levels are needed, requiring higher AAV doses, the hepatotoxicity and the inflammatory response of current vectors are both hurdles to be overcome [148,149,150]. It is particularly effective in recessive liver disorders without intrinsic hepatocyte damage. Long-term assessment of AAV gene therapy is important to assess whether loss of AAV genomes might compromise efficacy, both in adults as well as younger patients, in which liver growth is still ongoing [148]. Further development of other viral vector systems, such as lentiviral vectors that can integrate, may address some of these drawbacks. More recently developed technologies, such as genome editing, need to evolve to tools containing higher specificity with less off-target editing, achieve broader utility to address the heterogeneity of disease mutations, and preferably become smaller. Investigating off-target effects in preclinical studies in vivo has its limitations. Several in vitro gene therapy studies in human primary cells and in human HSCs did show off-target effects including chromosomal rearrangements, especially when introducing double-strand DNA breaks. Although strategies using single-strand nicks do appear to be safer, long-term careful monitoring of treated patients will be needed to identify adverse effects due to genotoxicity of in vivo gene editing and to reveal potential side effects [116,126,151,152]. Furthermore, genome editing will be a highly personalized approach for specific mutations, and costs need to be minimalized to be able to make these tailor-made approaches available to patients with inherited liver diseases.

## Figures and Tables

**Table 3:** An overview of pros and cons of AAV vectors and lentiviral vectors.

AAV Vectors	Lentiviral Vectors
Serotypes that can specifically target hepatocytes efficiently	No specific hepatocyte targeting
Typically nonintegrating (episomal)	Integrating into the genome
Potential liver toxicity at high doses	High transduction efficiency of liver is difficult
Stable viral particles	Typically unstable due to viral envelope
Packaging capacity up to 4.7 Kb	Packaging capacity up to 8 Kb
Prevalent pre-existing immunity in adult population	

**Table 4:** An overview of characteristics of ZFN, TALEN, and Crispr-Cas9.

	ZFN	TALEN	Crispr-Cas9
Mechanism	Artificial nucleases composed of zinc finger proteins that bind to the targeted DNA sequence and the nuclease domain of the Fokl restriction enzyme [94].	A transcription activator-like effector DNA-binding domain engineered to bind to a specific DNA sequence, which are fused with a nuclease cleavage domain to induce a double-strand DNA cut [96,103,104].	The Cas proteins induce a double-strand DNA cut and are directed by a guide RNA binding to a specific targeted DNA sequence [99].
	All induce a double-strand DNA break, activating DNA repair. Mostly repaired by nonhomologous end joining, causing small insertions or deletions, effectively eliminating expression of the encoded protein. In combination with an exogenous DNA repair template, inducing homology-directed repair (HDR), resulting in correction of pathologic mutations [105,106].		
Advantage	- high specificity (long recognition sequences) - mature technology - small (~1 kb)	- simple design compared to ZFN- high specificity and low off-target rate - can recognize longer DNA sequences	- precise and low off-target rate - time- and cost-saving
Disadvantage	- complex design - off-target effects - cytotoxicity	- large (~3 kb) - expensive	- requires a PAM site, restricting the versatility of genomic sequences that can be targeted - large (~4.2 kb)
Preclinical trials	Hemophilia B, Li et al., 2011 [107] Mucopolysaccharidosis type I, Ou et al., 2018 [108] Mucopolysaccharidosis type II, Laoharawee et al., 2018 [109]	α1-antitrypsin deficiency, Yusa et al., 2011 [110]	Hemophilia, Han et al., 2022 [111]

## References

[1] Nathwani A.C. (2022). Gene therapy for hemophilia. Hematol. Am. Soc. Hematol. Educ. Program..

[2] van Zwol W., van de Sluis B., Ginsberg H.N., Kuivenhoven J.A. (2024). VLDL Biogenesis and Secretion: It Takes a Village. Circ. Res..

[3] Scoditti E., Sabatini S., Carli F., Gastaldelli A. (2024). Hepatic glucose metabolism in the steatotic liver. Nat. Rev. Gastroenterol. Hepatol..

[4] Vimalesvaran S., Dhawan A. (2021). Liver transplantation for pediatric inherited metabolic liver diseases. World J. Hepatol..

[5] Montano-Loza A.J., Rodriguez-Peralvarez M.L., Pageaux G.P., Sanchez-Fueyo A., Feng S. (2023). Liver transplantation immunology: Immunosuppression, rejection, and immunomodulation. J. Hepatol..

[6] Nathwani A.C., McIntosh J., Sheridan R. (2022). Liver Gene Therapy. Hum. Gene Ther..

[7] Manno C.S., Pierce G.F., Arruda V.R., Glader B., Ragni M., Rasko J.J., Ozelo M.C., Hoots K., Blatt P., Konkle B. (2006). Successful transduction of liver in hemophilia by AAV-Factor IX and limitations imposed by the host immune response. Nat. Med..

[8] Nathwani A.C., Tuddenham E.G., Rangarajan S., Rosales C., McIntosh J., Linch D.C., Chowdary P., Riddell A., Pie A.J., Harrington C. (2011). Adenovirus-associated virus vector-mediated gene transfer in hemophilia B. N. Engl. J. Med..

[9] Mahlangu J., Kaczmarek R., von Drygalski A., Shapiro S., Chou S.C., Ozelo M.C., Kenet G., Peyvandi F., Wang M., Madan B. (2023). Two-Year Outcomes of Valoctocogene Roxaparvovec Therapy for Hemophilia A. N. Engl. J. Med..

[10] Castaman G., Coppens M., Pipe S.W. (2023). Etranacogene dezaparvovec for the treatment of adult patients with severe and moderately severe hemophilia B. Expert. Rev. Hematol..

[11] Flageul M., Aubert D., Pichard V., Nguyen T.H., Nowrouzi A., Schmidt M., Ferry N. (2009). Transient expression of genes delivered to newborn rat liver using recombinant adeno-associated virus 2/8 vectors. J. Gene Med..

[12] Shi X., Aronson S.J., Ten Bloemendaal L., Duijst S., Bakker R.S., de Waart D.R., Bortolussi G., Collaud F., Oude Elferink R.P., Muro A.F. (2021). Efficacy of AAV8-hUGT1A1 with Rapamycin in neonatal, suckling, and juvenile rats to model treatment in pediatric CNs patients. Mol. Ther. Methods Clin. Dev..

[13] Jiang H., Lillicrap D., Patarroyo-White S., Liu T., Qian X., Scallan C.D., Powell S., Keller T., McMurray M., Labelle A. (2006). Multiyear therapeutic benefit of AAV serotypes 2, 6, and 8 delivering factor VIII to hemophilia A mice and dogs. Blood.

[14] Bunting S., Zhang L., Xie L., Bullens S., Mahimkar R., Fong S., Sandza K., Harmon D., Yates B., Handyside B. (2018). Gene Therapy with BMN 270 Results in Therapeutic Levels of FVIII in Mice and Primates and Normalization of Bleeding in Hemophilic Mice. Mol. Ther..

[15] Pasi K.J., Rangarajan S., Mitchell N., Lester W., Symington E., Madan B., Laffan M., Russell C.B., Li M., Pierce G.F. (2020). Multiyear Follow-up of AAV5-hFVIII-SQ Gene Therapy for Hemophilia A. N. Engl. J. Med..

[16] George L.A., Monahan P.E., Eyster M.E., Sullivan S.K., Ragni M.V., Croteau S.E., Rasko J.E.J., Recht M., Samelson-Jones B.J., MacDougall A. (2021). Multiyear Factor VIII Expression after AAV Gene Transfer for Hemophilia A. N. Engl. J. Med..

[17] Madan B., Ozelo M.C., Raheja P., Symington E., Quon D.V., Leavitt A.D., Pipe S.W., Lowe G., Kenet G., Reding M.T. (2024). Three-year outcomes of valoctocogene roxaparvovec gene therapy for hemophilia A. J. Thromb. Haemost..

[18] Wang L., Takabe K., Bidlingmaier S.M., Ill C.R., Verma I.M. (1999). Sustained correction of bleeding disorder in hemophilia B mice by gene therapy. Proc. Natl. Acad. Sci. USA.

[19] Wang L., Nichols T.C., Read M.S., Bellinger D.A., Verma I.M. (2000). Sustained expression of therapeutic level of factor IX in hemophilia B dogs by AAV-mediated gene therapy in liver. Mol. Ther..

[20] Nathwani A.C., Gray J.T., Ng C.Y., Zhou J., Spence Y., Waddington S.N., Tuddenham E.G., Kemball-Cook G., McIntosh J., Boon-Spijker M. (2006). Self-complementary adeno-associated virus vectors containing a novel liver-specific human factor IX expression cassette enable highly efficient transduction of murine and nonhuman primate liver. Blood.

[21] Muczynski V., Nathwani A.C. (2024). AAV mediated gene therapy for haemophilia B: From the early attempts to modern trials. Thromb. Res..

[22] Seppen J., Bakker C., de Jong B., Kunne C., van den Oever K., Vandenberghe K., de Waart R., Twisk J., Bosma P. (2006). Adeno-associated virus vector serotypes mediate sustained correction of bilirubin UDP glucuronosyltransferase deficiency in rats. Mol. Ther..

[23] Bortolussi G., Zentilin L., Baj G., Giraudi P., Bellarosa C., Giacca M., Tiribelli C., Muro A.F. (2012). Rescue of bilirubin-induced neonatal lethality in a mouse model of Crigler-Najjar syndrome type I by AAV9-mediated gene transfer. FASEB J..

[24] D’Antiga L., Beuers U., Ronzitti G., Brunetti-Pierri N., Baumann U., Di Giorgio A., Aronson S., Hubert A., Romano R., Junge N. (2023). Gene Therapy in Patients with the Crigler-Najjar Syndrome. N. Engl. J. Med..

[25] Moscioni D., Morizono H., McCarter R.J., Stern A., Cabrera-Luque J., Hoang A., Sanmiguel J., Wu D., Bell P., Gao G.P. (2006). Long-term correction of ammonia metabolism and prolonged survival in ornithine transcarbamylase-deficient mice following liver-directed treatment with adeno-associated viral vectors. Mol. Ther..

[26] Cunningham S.C., Spinoulas A., Carpenter K.H., Wilcken B., Kuchel P.W., Alexander I.E. (2009). AAV2/8-mediated correction of OTC deficiency is robust in adult but not neonatal Spf(ash) mice. Mol. Ther..

[27] Cunningham S.C., Kok C.Y., Dane A.P., Carpenter K., Kizana E., Kuchel P.W., Alexander I.E. (2011). Induction and prevention of severe hyperammonemia in the spfash mouse model of ornithine transcarbamylase deficiency using shRNA and rAAV-mediated gene delivery. Mol. Ther..

[28] Wang L., Warzecha C.C., Kistner A., Chichester J.A., Bell P., Buza E.L., He Z., Pampena M.B., Couthouis J., Sethi S. (2022). Prednisolone reduces the interferon response to AAV in cynomolgus macaques and may increase liver gene expression. Mol. Ther. Methods Clin. Dev..

[29] Baruteau J., Cunningham S.C., Yilmaz B.S., Perocheau D.P., Eaglestone S., Burke D., Thrasher A.J., Waddington S.N., Lisowski L., Alexander I.E. (2021). Safety and efficacy of an engineered hepatotropic AAV gene therapy for ornithine transcarbamylase deficiency in cynomolgus monkeys. Mol. Ther. Methods Clin. Dev..

[30] De Sabbata G., Boisgerault F., Guarnaccia C., Iaconcig A., Bortolussi G., Collaud F., Ronzitti G., Sola M.S., Vidal P., Rouillon J. (2021). Long-term correction of ornithine transcarbamylase deficiency in Spf-Ash mice with a translationally optimized AAV vector. Mol. Ther. Methods Clin. Dev..

[31] Seker Yilmaz B., Gissen P. (2023). Genetic Therapy Approaches for Ornithine Transcarbamylase Deficiency. Biomedicines.

[32] Thomas J., Tan W.H., Khan A., Konczal L., Breilyn M.S., Hualde L.C., Couce M.L., Geberhiwot T., Guffon N., Harding C. (2024). Long-term safety and efficacy of DTX301 in adults with late-onset ornithine transcarbamylase (OTC) deficiency: A Phase 1/2 trial. Mol. Genet. Metab..

[33] Koeberl D.D., Sun B.D., Damodaran T.V., Brown T., Millington D.S., Benjamin D.K., Bird A., Schneider A., Hillman S., Jackson M. (2006). Early, sustained efficacy of adeno-associated virus vector-mediated gene therapy in glycogen storage disease type Ia. Gene Ther..

[34] Koeberl D.D., Pinto C., Sun B., Li S., Kozink D.M., Benjamin D.K., Demaster A.K., Kruse M.A., Vaughn V., Hillman S. (2008). AAV vector-mediated reversal of hypoglycemia in canine and murine glycogen storage disease type Ia. Mol. Ther..

[35] Watson G.L., Sayles J.N., Chen C., Elliger S.S., Elliger C.A., Raju N.R., Kurtzman G.J., Podsakoff G.M. (1998). Treatment of lysosomal storage disease in MPS VII mice using a recombinant adeno-associated virus. Gene Ther..

[36] Cardone M., Polito V.A., Pepe S., Mann L., D’Azzo A., Auricchio A., Ballabio A., Cosma M.P. (2006). Correction of Hunter syndrome in the MPSII mouse model by AAV2/8-mediated gene delivery. Hum. Mol. Genet..

[37] Cotugno G., Annunziata P., Tessitore A., O’Malley T., Capalbo A., Faella A., Bartolomeo R., O’Donnell P., Wang P., Russo F. (2011). Long-term amelioration of feline Mucopolysaccharidosis VI after AAV-mediated liver gene transfer. Mol. Ther..

[38] Hordeaux J., Hinderer C., Buza E.L., Louboutin J.P., Jahan T., Bell P., Chichester J.A., Tarantal A.F., Wilson J.M. (2019). Safe and Sustained Expression of Human Iduronidase After Intrathecal Administration of Adeno-Associated Virus Serotype 9 in Infant Rhesus Monkeys. Hum. Gene Ther..

[39] Rossi A., Brunetti-Pierri N. (2024). Gene therapies for mucopolysaccharidoses. J. Inherit. Metab. Dis..

[40] Unzu C., Sampedro A., Mauleon I., Alegre M., Beattie S.G., de Salamanca R.E., Snapper J., Twisk J., Petry H., Gonzalez-Aseguinolaza G. (2011). Sustained enzymatic correction by rAAV-mediated liver gene therapy protects against induced motor neuropathy in acute porphyria mice. Mol. Ther..

[41] Paneda A., Lopez-Franco E., Kaeppel C., Unzu C., Gil-Royo A.G., D’Avola D., Beattie S.G., Olague C., Ferrero R., Sampedro A. (2013). Safety and liver transduction efficacy of rAAV5-cohPBGD in nonhuman primates: A potential therapy for acute intermittent porphyria. Hum. Gene Ther..

[42] D’Avola D., Lopez-Franco E., Sangro B., Paneda A., Grossios N., Gil-Farina I., Benito A., Twisk J., Paz M., Ruiz J. (2016). Phase I open label liver-directed gene therapy clinical trial for acute intermittent porphyria. J. Hepatol..

[43] Li C., Xiao P., Gray S.J., Weinberg M.S., Samulski R.J. (2011). Combination therapy utilizing shRNA knockdown and an optimized resistant transgene for rescue of diseases caused by misfolded proteins. Proc. Natl. Acad. Sci. USA.

[44] Mueller C., Tang Q., Gruntman A., Blomenkamp K., Teckman J., Song L., Zamore P.D., Flotte T.R. (2012). Sustained miRNA-mediated knockdown of mutant AAT with simultaneous augmentation of wild-type AAT has minimal effect on global liver miRNA profiles. Mol. Ther..

[45] Bjursell M., Porritt M.J., Ericson E., Taheri-Ghahfarokhi A., Clausen M., Magnusson L., Admyre T., Nitsch R., Mayr L., Aasehaug L. (2018). Therapeutic Genome Editing with CRISPR/Cas9 in a Humanized Mouse Model Ameliorates alpha1-antitrypsin Deficiency Phenotype. eBioMedicine.

[46] Shen S., Sanchez M.E., Blomenkamp K., Corcoran E.M., Marco E., Yudkoff C.J., Jiang H., Teckman J.H., Bumcrot D., Albright C.F. (2018). Amelioration of Alpha-1 Antitrypsin Deficiency Diseases with Genome Editing in Transgenic Mice. Hum. Gene Ther..

[47] Flotte T.R., Brantly M.L., Spencer L.T., Byrne B.J., Spencer C.T., Baker D.J., Humphries M. (2004). Phase I trial of intramuscular injection of a recombinant adeno-associated virus alpha 1-antitrypsin (rAAV2-CB-hAAT) gene vector to AAT-deficient adults. Hum. Gene Ther..

[48] Brantly M.L., Spencer L.T., Humphries M., Conlon T.J., Spencer C.T., Poirier A., Garlington W., Baker D., Song S., Berns K.I. (2006). Phase I trial of intramuscular injection of a recombinant adeno-associated virus serotype 2 alphal-antitrypsin (AAT) vector in AAT-deficient adults. Hum. Gene Ther..

[49] Flotte T.R., Trapnell B.C., Humphries M., Carey B., Calcedo R., Rouhani F., Campbell-Thompson M., Yachnis A.T., Sandhaus R.A., McElvaney N.G. (2011). Phase 2 clinical trial of a recombinant adeno-associated viral vector expressing alpha1-antitrypsin: Interim results. Hum. Gene Ther..

[50] Strnad P., San Martin J. (2023). RNAi therapeutics for diseases involving protein aggregation: Fazirsiran for alpha-1 antitrypsin deficiency-associated liver disease. Expert. Opin. Investig. Drugs.

[51] Greig J.A., Nordin J.M.L., Smith M.K., Ashley S.N., Draper C., Zhu Y., Bell P., Buza E.L., Wilson J.M. (2019). A Gene Therapy Approach to Improve Copper Metabolism and Prevent Liver Damage in a Mouse Model of Wilson Disease. Hum. Gene Ther. Clin. Dev..

[52] Lebherz C., Gao G., Louboutin J.P., Millar J., Rader D., Wilson J.M. (2004). Gene therapy with novel adeno-associated virus vectors substantially diminishes atherosclerosis in a murine model of familial hypercholesterolemia. J. Gene Med..

[53] Greig J.A., Limberis M.P., Bell P., Chen S.J., Calcedo R., Rader D.J., Wilson J.M. (2017). Nonclinical Pharmacology/Toxicology Study of AAV8.TBG.mLDLR and AAV8.TBG.hLDLR in a Mouse Model of Homozygous Familial Hypercholesterolemia. Hum. Gene Ther. Clin. Dev..

[54] Wang L., Muthuramu I., Somanathan S., Zhang H., Bell P., He Z., Yu H., Zhu Y., Tretiakova A.P., Wilson J.M. (2021). Developing a second-generation clinical candidate AAV vector for gene therapy of familial hypercholesterolemia. Mol. Ther. Methods Clin. Dev..

[55] Greig J.A., Limberis M.P., Bell P., Chen S.J., Calcedo R., Rader D.J., Wilson J.M. (2017). Non-Clinical Study Examining AAV8.TBG.hLDLR Vector-Associated Toxicity in Chow-Fed Wild-Type and LDLR(+/−) Rhesus Macaques. Hum. Gene Ther. Clin. Dev..

[56] Aronson S.J., Bakker R.S., Shi X., Duijst S., Ten Bloemendaal L., de Waart D.R., Verheij J., Ronzitti G., Oude Elferink R.P., Beuers U. (2019). Liver-directed gene therapy results in long-term correction of progressive familial intrahepatic cholestasis type 3 in mice. J. Hepatol..

[57] Weber N.D., Odriozola L., Martinez-Garcia J., Ferrer V., Douar A., Benichou B., Gonzalez-Aseguinolaza G., Smerdou C. (2019). Gene therapy for progressive familial intrahepatic cholestasis type 3 in a clinically relevant mouse model. Nat. Commun..

[58] Weber N.D., Odriozola L., Ros-Ganan I., Garcia-Porrero G., Salas D., Argemi J., Combal J.P., Kishimoto T.K., Gonzalez-Aseguinolaza G. (2023). Rescue of infant progressive familial intrahepatic cholestasis type 3 mice by repeated dosing of AAV gene therapy. JHEP Rep..

[59] European Medicines Agency (2023). Roctavian.

[60] US Food and Drug Administration (2023). Roctavian.

[61] European Medicines Agency (2023). Hemgenix.

[62] US Food and Drug Administration HEMGENIX. https://www.fda.gov/vaccines-blood-biologics/vaccines/hemgenix.

[63] Bala N.S., Thornburg C.D. (2024). Gene Therapy in Hemophilia A: Achievements, Challenges, and Perspectives. Semin. Thromb. Hemost..

[64] Aronson S.J., Veron P., Collaud F., Hubert A., Delahais V., Honnet G., de Knegt R.J., Junge N., Baumann U., Di Giorgio A. (2019). Prevalence and Relevance of Pre-Existing Anti-Adeno-Associated Virus Immunity in the Context of Gene Therapy for Crigler-Najjar Syndrome. Hum. Gene Ther..

[65] Chhabra A., Bashirians G., Petropoulos C.J., Wrin T., Paliwal Y., Henstock P.V., Somanathan S., da Fonseca Pereira C., Winburn I., Rasko J.E.J. (2024). Global seroprevalence of neutralizing antibodies against adeno-associated virus serotypes used for human gene therapies. Mol. Ther. Methods Clin. Dev..

[66] Malato Y., Naqvi S., Schurmann N., Ng R., Wang B., Zape J., Kay M.A., Grimm D., Willenbring H. (2011). Fate tracing of mature hepatocytes in mouse liver homeostasis and regeneration. J. Clin. Investig..

[67] Bortolussi G., Zentillin L., Vanikova J., Bockor L., Bellarosa C., Mancarella A., Vianello E., Tiribelli C., Giacca M., Vitek L. (2014). Life-long correction of hyperbilirubinemia with a neonatal liver-specific AAV-mediated gene transfer in a lethal mouse model of Crigler-Najjar Syndrome. Hum. Gene Ther..

[68] Rana J., Herzog R.W., Munoz-Melero M., Yamada K., Kumar S.R.P., Lam A.K., Markusic D.M., Duan D., Terhorst C., Byrne B.J. (2024). B cell focused transient immune suppression protocol for efficient AAV readministration to the liver. Mol. Ther. Methods Clin. Dev..

[69] Perocheau D.P., Cunningham S., Lee J., Antinao Diaz J., Waddington S.N., Gilmour K., Eaglestone S., Lisowski L., Thrasher A.J., Alexander I.E. (2019). Age-Related Seroprevalence of Antibodies Against AAV-LK03 in a UK Population Cohort. Hum. Gene Ther..

[70] Leborgne C., Barbon E., Alexander J.M., Hanby H., Delignat S., Cohen D.M., Collaud F., Muraleetharan S., Lupo D., Silverberg J. (2020). IgG-cleaving endopeptidase enables in vivo gene therapy in the presence of anti-AAV neutralizing antibodies. Nat. Med..

[71] Bertin B., Veron P., Leborgne C., Deschamps J.Y., Moullec S., Fromes Y., Collaud F., Boutin S., Latournerie V., van Wittenberghe L. (2020). Capsid-specific removal of circulating antibodies to adeno-associated virus vectors. Sci. Rep..

[72] Seppen J., Bosma P.J., Goldhoorn B.G., Bakker C.T., Chowdhury J.R., Chowdhury N.R., Jansen P.L., Oude Elferink R.P. (1994). Discrimination between Crigler-Najjar type I and II by expression of mutant bilirubin uridine diphosphate-glucuronosyltransferase. J. Clin. Investg..

[73] (2020). High-dose AAV gene therapy deaths. Nat. Biotechnol..

[74] Paulk N.K., Pekrun K., Zhu E., Nygaard S., Li B., Xu J., Chu K., Leborgne C., Dane A.P., Haft A. (2018). Bioengineered AAV Capsids with Combined High Human Liver Transduction In Vivo and Unique Humoral Seroreactivity. Mol. Ther..

[75] Suriano C.M., Kumar N., Verpeut J.L., Ma J., Jung C., Dunn C.E., Carvajal B.V., Nguyen A.V., Boulanger L.M. (2024). An innate immune response to adeno-associated virus genomes decreases cortical dendritic complexity and disrupts synaptic transmission. Mol. Ther..

[76] Li X., Wei X., Lin J., Ou L. (2022). A versatile toolkit for overcoming AAV immunity. Front. Immunol..

[77] Xiang Z., Kurupati R.K., Li Y., Kuranda K., Zhou X., Mingozzi F., High K.A., Ertl H.C.J. (2020). The Effect of CpG Sequences on Capsid-Specific CD8(+) T Cell Responses to AAV Vector Gene Transfer. Mol. Ther..

[78] Duan D., Yue Y., Engelhardt J.F. (2001). Expanding AAV packaging capacity with trans-splicing or overlapping vectors: A quantitative comparison. Mol. Ther..

[79] Zufferey R., Dull T., Mandel R.J., Bukovsky A., Quiroz D., Naldini L., Trono D. (1998). Self-inactivating lentivirus vector for safe and efficient in vivo gene delivery. J. Virol..

[80] Dull T., Zufferey R., Kelly M., Mandel R.J., Nguyen M., Trono D., Naldini L. (1998). A third-generation lentivirus vector with a conditional packaging system. J. Virol..

[81] Kao V.Y., Ferreira S., Waddington S.N., Antoniou M.N. (2016). Haemophilia B curative FIX production from a low dose UCOE-based lentiviral vector following hepatic pre-natal delivery. Curr. Gene Ther..

[82] Nicolas C.T., VanLith C.J., Hickey R.D., Du Z., Hillin L.G., Guthman R.M., Cao W.J., Haugo B., Lillegard A., Roy D. (2022). In Vivo lentiviral vector gene therapy to cure hereditary tyrosinemia type 1 and prevent development of precancerous and cancerous lesions. Nat. Commun..

[83] Cantore A., Ranzani M., Bartholomae C.C., Volpin M., Valle P.D., Sanvito F., Sergi L.S., Gallina P., Benedicenti F., Bellinger D. (2015). Liver-directed lentiviral gene therapy in a dog model of hemophilia B. Sci. Transl. Med..

[84] Mount J.D., Herzog R.W., Tillson D.M., Goodman S.A., Robinson N., McCleland M.L., Bellinger D., Nichols T.C., Arruda V.R., Lothrop C.D. (2002). Sustained phenotypic correction of hemophilia B dogs with a factor IX null mutation by liver-directed gene therapy. Blood.

[85] Milani M., Canepari C., Liu T., Biffi M., Russo F., Plati T., Curto R., Patarroyo-White S., Drager D., Visigalli I. (2022). Liver-directed lentiviral gene therapy corrects hemophilia A mice and achieves normal-range factor VIII activity in non-human primates. Nat. Commun..

[86] Milani M., Annoni A., Moalli F., Liu T., Cesana D., Calabria A., Bartolaccini S., Biffi M., Russo F., Visigalli I. (2019). Phagocytosis-shielded lentiviral vectors improve liver gene therapy in nonhuman primates. Sci. Transl. Med..

[87] De Vree J.M., Ottenhoff R., Bosma P.J., Smith A.J., Aten J., Oude Elferink R.P. (2000). Correction of liver disease by hepatocyte transplantation in a mouse model of progressive familial intrahepatic cholestasis. Gastroenterology.

[88] van Til N.P., Heutinck K.M., van der Rijt R., Paulusma C.C., van Wijland M., Markusic D.M., Elferink R.P., Seppen J. (2008). Alteration of viral lipid composition by expression of the phospholipid floppase ABCB4 reduces HIV vector infectivity. Retrovirology.

[89] Brady T., Bushman F.D. (2011). Nondividing cells: A safer bet for integrating vectors?. Mol. Ther..

[90] Matrai J., Cantore A., Bartholomae C.C., Annoni A., Wang W., Acosta-Sanchez A., Samara-Kuko E., De Waele L., Ma L., Genovese P. (2011). Hepatocyte-targeted expression by integrase-defective lentiviral vectors induces antigen-specific tolerance in mice with low genotoxic risk. Hepatology.

[91] Tucci F., Galimberti S., Naldini L., Valsecchi M.G., Aiuti A. (2022). A systematic review and meta-analysis of gene therapy with hematopoietic stem and progenitor cells for monogenic disorders. Nat. Commun..

[92] Eichler F., Duncan C., Musolino P.L., Orchard P.J., De Oliveira S., Thrasher A.J., Armant M., Dansereau C., Lund T.C., Miller W.P. (2017). Hematopoietic Stem-Cell Gene Therapy for Cerebral Adrenoleukodystrophy. N. Engl. J. Med..

[93] Hickey R.D., Nicolas C.T., Allen K., Mao S., Elgilani F., Glorioso J., Amiot B., VanLith C., Guthman R., Du Z. (2019). Autologous Gene and Cell Therapy Provides Safe and Long-Term Curative Therapy in A Large Pig Model of Hereditary Tyrosinemia Type 1. Cell Transplant..

[94] Kim Y.G., Cha J., Chandrasegaran S. (1996). Hybrid restriction enzymes: Zinc finger fusions to Fok I cleavage domain. Proc. Natl. Acad. Sci. USA.

[95] Epinat J.C., Arnould S., Chames P., Rochaix P., Desfontaines D., Puzin C., Patin A., Zanghellini A., Paques F., Lacroix E. (2003). A novel engineered meganuclease induces homologous recombination in yeast and mammalian cells. Nucleic Acids Res..

[96] Christian M., Cermak T., Doyle E.L., Schmidt C., Zhang F., Hummel A., Bogdanove A.J., Voytas D.F. (2010). Targeting DNA double-strand breaks with TAL effector nucleases. Genetics.

[97] Jinek M., Chylinski K., Fonfara I., Hauer M., Doudna J.A., Charpentier E. (2012). A programmable dual-RNA-guided DNA endonuclease in adaptive bacterial immunity. Science.

[98] Cong L., Ran F.A., Cox D., Lin S., Barretto R., Habib N., Hsu P.D., Wu X., Jiang W., Marraffini L.A. (2013). Multiplex genome engineering using CRISPR/Cas systems. Science.

[99] Gasiunas G., Barrangou R., Horvath P., Siksnys V. (2012). Cas9-crRNA ribonucleoprotein complex mediates specific DNA cleavage for adaptive immunity in bacteria. Proc. Natl. Acad. Sci. USA.

[100] Wiedenheft B., Lander G.C., Zhou K., Jore M.M., Brouns S.J.J., van der Oost J., Doudna J.A., Nogales E. (2011). Structures of the RNA-guided surveillance complex from a bacterial immune system. Nature.

[101] Hsu P.D., Lander E.S., Zhang F. (2014). Development and applications of CRISPR-Cas9 for genome engineering. Cell.

[102] Pickar-Oliver A., Gersbach C.A. (2019). The next generation of CRISPR-Cas technologies and applications. Nat. Rev. Mol. Cell Biol..

[103] Boch J., Scholze H., Schornack S., Landgraf A., Hahn S., Kay S., Lahaye T., Nickstadt A., Bonas U. (2009). Breaking the code of DNA binding specificity of TAL-type III effectors. Science.

[104] Moscou M.J., Bogdanove A.J. (2009). A simple cipher governs DNA recognition by TAL effectors. Science.

[105] Gaj T., Gersbach C.A., Barbas C.F. (2013). ZFN, TALEN, and CRISPR/Cas-based methods for genome engineering. Trends Biotechnol..

[106] Carroll D. (2011). Genome engineering with zinc-finger nucleases. Genetics.

[107] Li H., Haurigot V., Doyon Y., Li T., Wong S.Y., Bhagwat A.S., Malani N., Anguela X.M., Sharma R., Ivanciu L. (2011). In Vivo genome editing restores haemostasis in a mouse model of haemophilia. Nature.

[108] Ou L., DeKelver R.C., Rohde M., Tom S., Radeke R., St Martin S.J., Santiago Y., Sproul S., Przybilla M.J., Koniar B.L. (2019). ZFN-Mediated In Vivo Genome Editing Corrects Murine Hurler Syndrome. Mol. Ther..

[109] Laoharawee K., DeKelver R.C., Podetz-Pedersen K.M., Rohde M., Sproul S., Nguyen H.O., Nguyen T., St Martin S.J., Ou L., Tom S. (2018). Dose-Dependent Prevention of Metabolic and Neurologic Disease in Murine MPS II by ZFN-Mediated In Vivo Genome Editing. Mol. Ther..

[110] Yusa K., Rashid S.T., Strick-Marchand H., Varela I., Liu P.Q., Paschon D.E., Miranda E., Ordonez A., Hannan N.R., Rouhani F.J. (2011). Targeted gene correction of alpha1-antitrypsin deficiency in induced pluripotent stem cells. Nature.

[111] Han J.P., Kim M., Choi B.S., Lee J.H., Lee G.S., Jeong M., Lee Y., Kim E.A., Oh H.K., Go N. (2022). In Vivo delivery of CRISPR-Cas9 using lipid nanoparticles enables antithrombin gene editing for sustainable hemophilia A and B therapy. Sci. Adv..

[112] Patsali P., Mussolino C., Ladas P., Floga A., Kolnagou A., Christou S., Sitarou M., Antoniou M.N., Cathomen T., Lederer C.W. (2019). The Scope for Thalassemia Gene Therapy by Disruption of Aberrant Regulatory Elements. J. Clin. Med..

[113] Ottaviano G., Qasim W. (2022). Genome-Edited T Cell Therapies. Hematol. Oncol. Clin. N. Am..

[114] Monteys A.M., Ebanks S.A., Keiser M.S., Davidson B.L. (2017). CRISPR/Cas9 Editing of the Mutant Huntingtin Allele In Vitro and In Vivo. Mol. Ther..

[115] Longhurst H.J., Lindsay K., Petersen R.S., Fijen L.M., Gurugama P., Maag D., Butler J.S., Shah M.Y., Golden A., Xu Y. (2024). CRISPR-Cas9 In Vivo Gene Editing of KLKB1 for Hereditary Angioedema. N. Engl. J. Med..

[116] Turchiano G., Andrieux G., Klermund J., Blattner G., Pennucci V., El Gaz M., Monaco G., Poddar S., Mussolino C., Cornu T.I. (2021). Quantitative evaluation of chromosomal rearrangements in gene-edited human stem cells by CAST-Seq. Cell Stem Cell.

[117] Torella L., Klermund J., Bilbao-Arribas M., Tamayo I., Andrieux G., Chmielewski K.O., Vales A., Olague C., Moreno-Luqui D., Raimondi I. (2024). Efficient and safe therapeutic use of paired Cas9-nickases for primary hyperoxaluria type 1. EMBO Mol. Med..

[118] Jung C.J., Zhang J., Trenchard E., Lloyd K.C., West D.B., Rosen B., de Jong P.J. (2017). Efficient gene targeting in mouse zygotes mediated by CRISPR/Cas9-protein. Transgenic Res..

[119] Song C.Q., Wang D., Jiang T., O’Connor K., Tang Q., Cai L., Li X., Weng Z., Yin H., Gao G. (2018). In Vivo Genome Editing Partially Restores Alpha1-Antitrypsin in a Murine Model of AAT Deficiency. Hum. Gene Ther..

[120] Lisjak M., De Caneva A., Marais T., Barbon E., Biferi M.G., Porro F., Barzel A., Zentilin L., Kay M.A., Mingozzi F. (2022). Promoterless Gene Targeting Approach Combined to CRISPR/Cas9 Efficiently Corrects Hemophilia B Phenotype in Neonatal Mice. Front. Genome Ed..

[121] Sharma R., Anguela X.M., Doyon Y., Wechsler T., DeKelver R.C., Sproul S., Paschon D.E., Miller J.C., Davidson R.J., Shivak D. (2015). In Vivo genome editing of the albumin locus as a platform for protein replacement therapy. Blood.

[122] Barzel A., Paulk N.K., Shi Y., Huang Y., Chu K., Zhang F., Valdmanis P.N., Spector L.P., Porteus M.H., Gaensler K.M. (2015). Promoterless gene targeting without nucleases ameliorates haemophilia B in mice. Nature.

[123] Porro F., Bortolussi G., Barzel A., De Caneva A., Iaconcig A., Vodret S., Zentilin L., Kay M.A., Muro A.F. (2017). Promoterless gene targeting without nucleases rescues lethality of a Crigler-Najjar syndrome mouse model. EMBO Mol. Med..

[124] Harmatz P., Prada C.E., Burton B.K., Lau H., Kessler C.M., Cao L., Falaleeva M., Villegas A.G., Zeitler J., Meyer K. (2022). First-in-human in vivo genome editing via AAV-zinc-finger nucleases for mucopolysaccharidosis I/II and hemophilia B. Mol. Ther..

[125] Tsuji S., Stephens C.J., Bortolussi G., Zhang F., Baj G., Jang H., de Alencastro G., Muro A.F., Pekrun K., Kay M.A. (2022). Fludarabine increases nuclease-free AAV- and CRISPR/Cas9-mediated homologous recombination in mice. Nat. Biotechnol..

[126] Klermund J., Rhiel M., Kocher T., Chmielewski K.O., Bischof J., Andrieux G., El Gaz M., Hainzl S., Boerries M., Cornu T.I. (2024). On- and off-target effects of paired CRISPR-Cas nickase in primary human cells. Mol. Ther..

[127] Nunez J.K., Chen J., Pommier G.C., Cogan J.Z., Replogle J.M., Adriaens C., Ramadoss G.N., Shi Q., Hung K.L., Samelson A.J. (2021). Genome-wide programmable transcriptional memory by CRISPR-based epigenome editing. Cell.

[128] Matsoukas I.G. (2020). Prime Editing: Genome Editing for Rare Genetic Diseases Without Double-Strand Breaks or Donor DNA. Front. Genet..

[129] Rattananon P., Anurathapan U., Bhukhai K., Hongeng S. (2021). The Future of Gene Therapy for Transfusion-Dependent Beta-Thalassemia: The Power of the Lentiviral Vector for Genetically Modified Hematopoietic Stem Cells. Front. Pharmacol..

[130] Liu P., Liang S.Q., Zheng C., Mintzer E., Zhao Y.G., Ponnienselvan K., Mir A., Sontheimer E.J., Gao G., Flotte T.R. (2021). Improved prime editors enable pathogenic allele correction and cancer modelling in adult mice. Nat. Commun..

[131] Rees H.A., Liu D.R. (2018). Base editing: Precision chemistry on the genome and transcriptome of living cells. Nat. Rev. Genet..

[132] Levy J.M., Yeh W.H., Pendse N., Davis J.R., Hennessey E., Butcher R., Koblan L.W., Comander J., Liu Q., Liu D.R. (2020). Cytosine and adenine base editing of the brain, liver, retina, heart and skeletal muscle of mice via adeno-associated viruses. Nat. Biomed. Eng..

[133] Slesarenko Y.S., Lavrov A.V., Smirnikhina S.A. (2022). Off-target effects of base editors: What we know and how we can reduce it. Curr. Genet..

[134] Song C.Q., Jiang T., Richter M., Rhym L.H., Koblan L.W., Zafra M.P., Schatoff E.M., Doman J.L., Cao Y., Dow L.E. (2020). Adenine base editing in an adult mouse model of tyrosinaemia. Nat. Biomed. Eng..

[135] Rothgangl T., Dennis M.K., Lin P.J.C., Oka R., Witzigmann D., Villiger L., Qi W., Hruzova M., Kissling L., Lenggenhager D. (2021). In Vivo adenine base editing of PCSK9 in macaques reduces LDL cholesterol levels. Nat. Biotechnol..

[136] Packer M.S., Chowdhary V., Lung G., Cheng L.I., Aratyn-Schaus Y., Leboeuf D., Smith S., Shah A., Chen D., Zieger M. (2022). Evaluation of cytosine base editing and adenine base editing as a potential treatment for alpha-1 antitrypsin deficiency. Mol. Ther..

[137] Davis J.R., Wang X., Witte I.P., Huang T.P., Levy J.M., Raguram A., Banskota S., Seidah N.G., Musunuru K., Liu D.R. (2022). Efficient in vivo base editing via single adeno-associated viruses with size-optimized genomes encoding compact adenine base editors. Nat. Biomed. Eng..

[138] Hou X., Zaks T., Langer R., Dong Y. (2021). Lipid nanoparticles for mRNA delivery. Nat. Rev. Mater..

[139] Villiger L., Rothgangl T., Witzigmann D., Oka R., Lin P.J.C., Qi W., Janjuha S., Berk C., Ringnalda F., Beattie M.B. (2021). In Vivo cytidine base editing of hepatocytes without detectable off-target mutations in RNA and DNA. Nat. Biomed. Eng..

[140] Anzalone A.V., Randolph P.B., Davis J.R., Sousa A.A., Koblan L.W., Levy J.M., Chen P.J., Wilson C., Newby G.A., Raguram A. (2019). Search-and-replace genome editing without double-strand breaks or donor DNA. Nature.

[141] Zhao Z., Shang P., Mohanraju P., Geijsen N. (2023). Prime editing: Advances and therapeutic applications. Trends Biotechnol..

[142] Davis J.R., Banskota S., Levy J.M., Newby G.A., Wang X., Anzalone A.V., Nelson A.T., Chen P.J., Hennes A.D., An M. (2024). Efficient prime editing in mouse brain, liver and heart with dual AAVs. Nat. Biotechnol..

[143] Bock D., Rothgangl T., Villiger L., Schmidheini L., Matsushita M., Mathis N., Ioannidi E., Rimann N., Grisch-Chan H.M., Kreutzer S. (2022). In Vivo prime editing of a metabolic liver disease in mice. Sci. Transl. Med..

[144] Brooks D.L., Whittaker M.N., Qu P., Musunuru K., Ahrens-Nicklas R.C., Wang X. (2023). Efficient in vivo prime editing corrects the most frequent phenylketonuria variant, associated with high unmet medical need. Am. J. Hum. Genet..

[145] Brooks D.L., Carrasco M.J., Qu P., Peranteau W.H., Ahrens-Nicklas R.C., Musunuru K., Alameh M.G., Wang X. (2023). Rapid and definitive treatment of phenylketonuria in variant-humanized mice with corrective editing. Nat. Commun..

[146] Chen P.J., Liu D.R. (2023). Prime editing for precise and highly versatile genome manipulation. Nat. Rev. Genet..

[147] De Caneva A., Porro F., Bortolussi G., Sola R., Lisjak M., Barzel A., Giacca M., Kay M.A., Vlahovicek K., Zentilin L. (2019). Coupling AAV-mediated promoterless gene targeting to SaCas9 nuclease to efficiently correct liver metabolic diseases. JCI Insight.

[148] Zabaleta N., Unzu C., Weber N.D., Gonzalez-Aseguinolaza G. (2023). Gene therapy for liver diseases—Progress and challenges. Nat. Rev. Gastroenterol. Hepatol..

[149] Baruteau J., Brunetti-Pierri N., Gissen P. (2024). Liver-directed gene therapy for inherited metabolic diseases. J. Inherit. Metab. Dis..

[150] Ghasemzad M., Hashemi M., Lavasani Z.M., Hossein-Khannazer N., Bakhshandeh H., Gramignoli R., Keshavarz Alikhani H., Najimi M., Nikeghbalian S., Vosough M. (2022). Novel Gene-Correction-Based Therapeutic Modalities for Monogenic Liver Disorders. Bioengineering.

[151] Raimondi F., Siow K.M., Wrona D., Fuster-Garcia C., Pastukhov O., Schmitz M., Bargsten K., Kissling L., Swarts D.C., Andrieux G. (2024). Gene editing of NCF1 loci is associated with homologous recombination and chromosomal rearrangements. Commun. Biol..

[152] Frati G., Brusson M., Sartre G., Mlayah B., Felix T., Chalumeau A., Antoniou P., Hardouin G., Concordet J.P., Romano O. (2024). Safety and efficacy studies of CRISPR-Cas9 treatment of sickle cell disease highlights disease-specific responses. Mol. Ther..

